# Effect of Lipoxin A_4_ on Lipopolysaccharide-Induced Endothelial Hyperpermeability

**DOI:** 10.1100/tsw.2011.98

**Published:** 2011-05-05

**Authors:** Huayan Pang, Pan Yi, Ping Wu, Zhuoya Liu, Zhongjie Liu, Jianming Gong, Hua Hao, Lei Cai, Duyun Ye, Yinping Huang

**Affiliations:** ^1^ Department of Obstetrics and Gynecology, First Affiliated Hospital of Wenzhou Medical College, Wenzhou, Zhejiang, China; ^2^ Department of Pathophysiology, Tongji Medical College, Huazhong University of Science and Technology, Wuhan, Hubei, China

**Keywords:** lipoxin, endothelial permeability, pre-eclampsia, VE-cadherin, Nrf2, oxidative stress

## Abstract

Excessive oxidative stress, decreased antioxidant capacity, and enhanced cellular calcium levels are initial factors that cause endothelial cell (EC) hyperpermeability, which represents a crucial event in the pathogenesis of pre-eclampsia. Lipoxin A4 (LXA_4_) strongly attenuated lipopolysaccharide (LPS)-induced hyperpermeability through maintaining the normal expression of VE-cadherin and β-catenin. This effect was mainly mediated by a specific LXA_4_ receptor. LXA_4_ could also obviously inhibit LPS-induced elevation of the cellular calcium level and up-regulation of the transient receptor potential protein family C 1, an important calcium channel in ECs. At the same time, LXA_4_ strongly blocked LPS-triggered reactive oxidative species production, while it promoted the expression of the NF-E2 related factor 2 (Nrf2) protein. Our findings demonstrate that LXA_4_ could prevent the EC hyperpermeability induced by LPS in human umbilical vein endothelial cells (HUVECs), under which the possible mechanism is through Nrf2 as well as Ca^2+^-sensitive pathways.

### INTRODUCTION

Pre-eclampsia (PE), a pregnancy-specific syndrome characterized by the onset of hypertension, proteinuria, and edema, has been reported in approximately 8% of all pregnancies[1]. Although the pathophysiology of PE still remains undefined, placental oxidative stress is regarded as a key event[1,2,3]. It is widely accepted that in pregnant women with PE, excessive generation of reactive oxidative species (ROS) in the placenta contributes to widespread maternal vascular endothelial cell (EC) hyperpermeability[2].

Lipoxins (LXs) are the first identified lipid family serving as endogenous "braking signals" in inflammation that possesses a wide spectrum of anti-inflammatory and proresolution bioactions[4]. Recently, we observed that PE patients presented much lower LX levels in systemic circulation (data not shown). In addition, we previously confirmed that LXs had an antioxidant effect both in lipopolysaccharide (LPS)-stimulated macrophages[5,6] and in carbon tetrachloride–induced liver injury[7]. Combining these above results, we speculate that deficient LX production might conduce to persistent inflammatory and oxidative conditions, which in turn cause EC hyperpermeability, but the detailed mechanism is still unknown.

Therefore, the current study aimed to elucidate the role of lipoxin $A_4$ ($LXA_4$), a major endogenous LX, on preventing LPS-induced endothelial barrier disruption in human umbilical vein endothelial cells (HUVECs), which is a commonly applied cell model for studying systemic vascular function[8]. To further explore the possible mechanism, cellular calcium and NF-E2 related factor 2 (Nrf2), a crucial transcription factor in regulating cellular antioxidant response and phase II enzymes[9], were also investigated.

### MATERIALS AND METHODS

- **Materials** — $LXA_4$ was from Cayman Chemical (USA) and was stored at –80°C until diluted in serum-free culture medium immediately before use. Commercially obtained $LXA_4$ was diluted in ethanol, of which the final concentration in the cell culture medium was 0.0036% or lower. Endothelial cell growth medium-2 (EGM-2) and fetal bovine serum (FBS) were purchased from GIBCO (Australia). LPS, bovine serum albumin (BSA), and Fura-2 acetoxymethyl ester (Fura-2/AM) were from Sigma Aldrich (USA). 2´,7´-Dichlorofluorescin diacetate (DCFH-DA) was from Molecular Probes (USA). Antibodies to VE-cadherin, β-catenin, Nrf-2, and β-actin were from Santa Cruz (USA). TRIzol Reagent, Moloney murine leukemia virus (M-MLV) reverse transcriptase, and Amplex Red Hydrogen Peroxide/Peroxidase Assay kit were from Invitrogen Life Technologies (USA). Horseradish peroxidase (HRP) or fluorescein isothiocyanate (FITC) conjugated secondary antibodies were from Pierce (USA).
- **Primary culture of HUVECs and treatments** — Human umbilical cords were obtained from women with normal pregnancies immediately after delivery from the First Affiliated Hospital of Wenzhou Medical College, which received permission from the local Ethics Committee. Primary HUVECs were prepared from umbilical veins and cultured as published previously[10]. Cells at passage 3–5 were cultured in EGM and 20% FBS at 37°C with 5% $CO_2$. Cells were serum starved for 24 h before experiments, and then treated with 10 µg/ml LPS with or without 100 nM $LXA_4$ for 12 h unless stated otherwise.
- **Measurement of endothelial permeability** — HUVECs ($1 \times 10^5/cm^2$) were plated on 1% gelatin-coated transwell inserts in 12-well plates until confluent. Endothelial permeability was determined with permeability coefficient of albumin (Pa) according to published method[11].
- **Morphological observation** — HUVECs were observed under an inverted microscope after regular hematoxylin and eosin (H&E) staining.
- **Immunofluorescence assay** — Immunofluorescence assay was performed to detect VE-cadherin according to our published method[12].
- **Western-blotting analysis** — Western blotting was performed to detect VE-cadherin, β-catenin, and Nrf-2 expression as described previously[6].
- **Reverse transcription – polymerase chain reaction (RT-PCR)** — Total RNA extraction, reverse transcription, and PCR were conducted according to the manuscript. Glyceraldehyde 3-phosphate dehydrogenase (GAPDH) served as internal control. Sequences of primers and reactions conditions are shown in the Supplementary material.
- **Measurement of cytosolic free calcium concentration ($[Ca^{2+}]i$)** — After cells were treated with LPS with or without $LXA_4$ for 6 h, $[Ca^{2+}]i$ was measured with Fura-2 according to our previous report[13]. The intensities of fluorescence due to excitation at 340 (F340) and 380

(F380) nm were measured after background subtraction, and F340/F380 was used to represent changes in $[Ca^{2+}]i$.

- **Intracellular ROS measurement —** Intracellular ROS levels were determined by measuring the oxidative conversion of DCFH-DA to fluorescent compound DCFH[14]. The results were expressed as the percentage of fluorescence intensity in control cells.
- **Measurement of $H_2O_2$ production by HUVECs —** The assay is based on the detection of $H_2O_2$ that reacts with Amplex Red in the presence of HRP with a 1:1 stoichiometry producing resorufin[15]. Amplex Red (50 μM) and HRP (5 U/2 ml) were added to cells exposed to LPS or $LXA_4$ for indicated periods of time. Fluorescence was detected at 37°C in a fluorescence spectrophotometer 30, 60, or 90 min after incubation. The excitation and emission wavelengths were 550 and 585 nm, respectively. Calibration signals were generated using known amounts of $H_2O_2$.
- **Statistical analysis —** Statistical analysis was performed using SPSS 13.0 for Windows. Data were presented as mean ±SEM and analyzed by Student's t test. Values of $p < 0.05$ indicated a statistically significant difference.

### RESULTS

#### $LXA_4$ Prevented LPS-Induced Hyperpermeability of HUVECs

After primary HUVECs were successfully isolated and cultured, which was confirmed by CD31 and von Willebrand factor (vWF) positive staining as shown in Supplementary Fig. 1, vascular permeability was measured. As presented in Fig. 1, exposure to LPS for 12 h markedly increased the Pa value to ~183% that of control cells, while coadministration of $LXA_4$ attenuated this LPS-induced hyperpermeability ($p <$ 0.05). It was also observed that 50–200 nM $LXA_4$ showed no dose-dependent manner. Therefore, in the following experiments, only 100 nM $LXA_4$ was applied.

Since the bioactions of $LXA_4$ were mainly elicited through the $LXA_4$ receptor (ALXR/FPRL-1), which had already been confirmed to exist in HUVECs[16], then 10 μM Boc-2 (an effective antagonist of FPRL-1) was used to explore the effect of FPRL-1. Results showed that when Boc-2 was administrated 30 min in advance, it effectively blocked the influence of $LXA_4$ on LPS-induced Pa enhancement ($p <$ 0.01; shown in Fig.1).

#### $LXA_4$ Maintained the Normal Morphology of HUVECs

The morphology of ECs could also reflect the cell contractile state and vascular permeability, thus it was observed with routine H&E staining. As shown in Fig. 2, cells in control groups showed a cobblestone-like morphology, with tight cell-cell contact. When treated with LPS for 12 h, HUVECs presented a thinner and flatter, spindle-shaped morphology. This morphological change was concomitant with the increased albumin permeability shown in Fig. 1. Meanwhile, shapes of cells cotreated with $LXA_4$ were well preserved and the cell-cell conjunction was recuperated. $LXA_4$ alone showed no effect on the morphology of HUVECs.

#### $LXA_4$ Maintained the Expression of VE-Cadherin and β-Catenin in LPS-Stimulated HUVECs

As normal expression of VE-cadherin and β-catenin is required for the maintenance of proper endothelial adhesion junctions (AJs) and vascular permeability[17], we further determined the role of these two proteins in the protective effect of $LXA_4$ on EC permeability. As shown in Fig. 3, both VE-cadherin mRNA

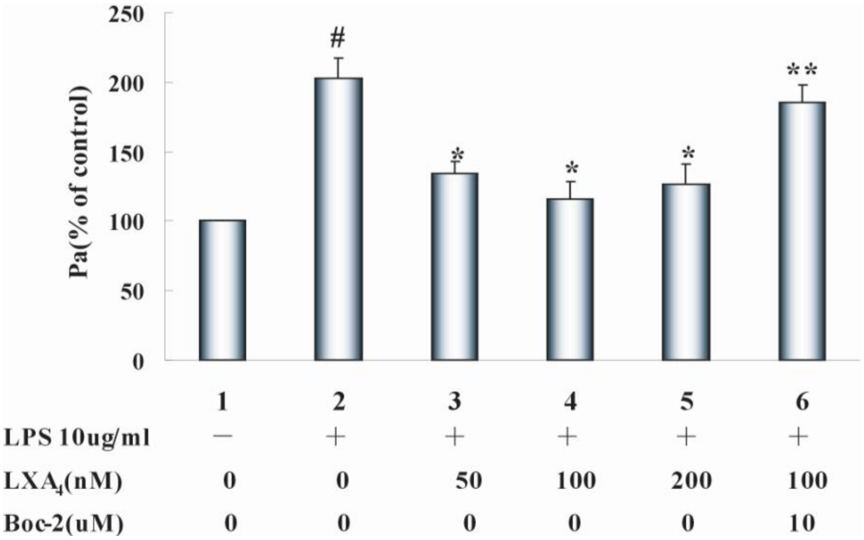

**FIGURE 1.** Effect of $LXA_4$ on endothelial permeability in LPS-stimulated HUVECs. LPS with or without $LXA_4$ were administrated for 12 h in the upper chamber. Boc-2 was applied 30 min prior to $LXA_4$. Pa value was applied to indicate the endothelial permeability. Data are expressed as percentage of control cells; #$p < 0.01$ compared to control cells; *$p < 0.05$ compared to LPS-stimulated cells; **$p < 0.01$ compared to $LXA_4$ + LPS–treated cells. All experiments were carried out in triplicate and for three independent times.

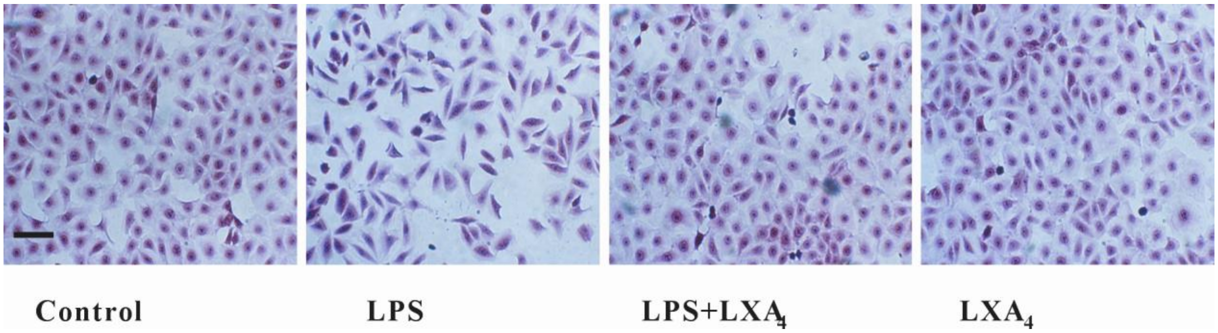

**FIGURE 2.** $LXA_4$ maintained the normal morphology of HUVECs. Representative images were from one of three separate experiments that yielded similar results.

and protein could be detectable in all of the four groups, with obviously the lowest level in cells treated with LPS ($p < 0.05$ vs. control cells or cells treated with both LPS and $LXA_4$). The Western blotting result was further confirmed by immunofluorescence assay with FITC-conjugated antibody to VE-cadherin. HUVECs in the control group demonstrated tight apposition between cells and strong membrane fluorescence. When LPS was administered, large intercellular gaps could be observed. At the same time, VE-cadherin staining was much lower. In cells cotreated with $LXA_4$, LPS-induced changes mentioned above, like gap formation and down-regulation of VE-cadherin, were much less apparent (Fig. 4). $LXA_4$ alone had no significant effect on VE-cadherin expression.

It was presented in Fig. 3 that $LXA_4$ blocked LPS-induced β-catenin protein down-regulation ($p <$ 0.05), while it showed no statistical effect on β-catenin protein expression in control cells.

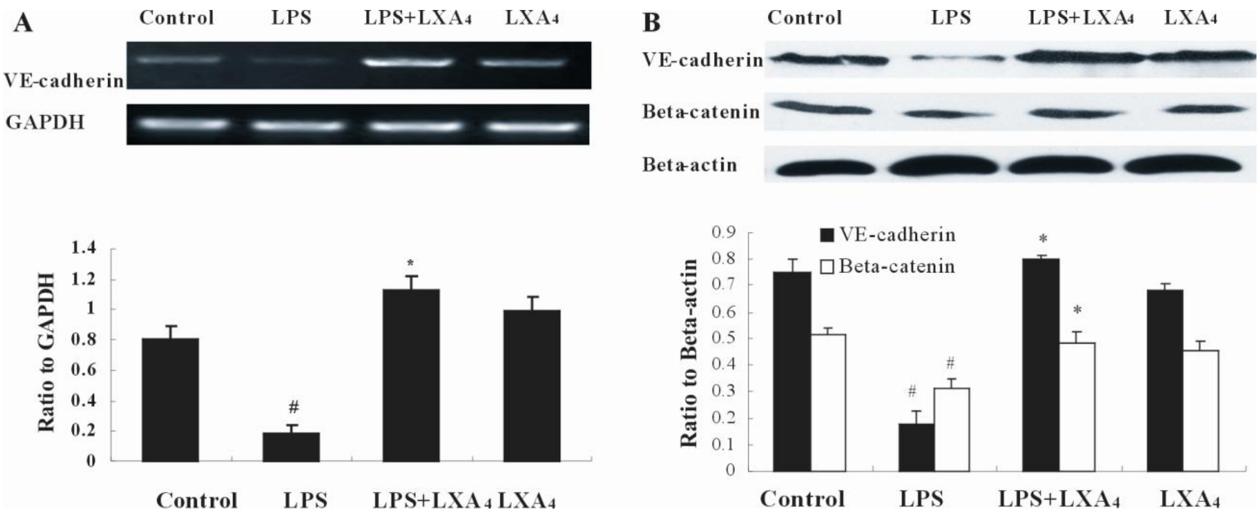

**FIGURE 3.** The expression of VE-cadherin and β-catenin in HUVECs under different conditions. (A) Representative RT-PCR for VE-cadherin is shown. GAPDH served as internal control. Bar graphs indicate means ± SEM from at least three independent experiments. (B) Representative Western blots for VE-cadherin and β-catenin are shown. Beta-actin served as internal control. Bar graphs indicate means ± SEM from at least three independent experiments; #$p < 0.05$ compared to control cells; *$p < 0.05$ compared to cells treated by LPS alone.

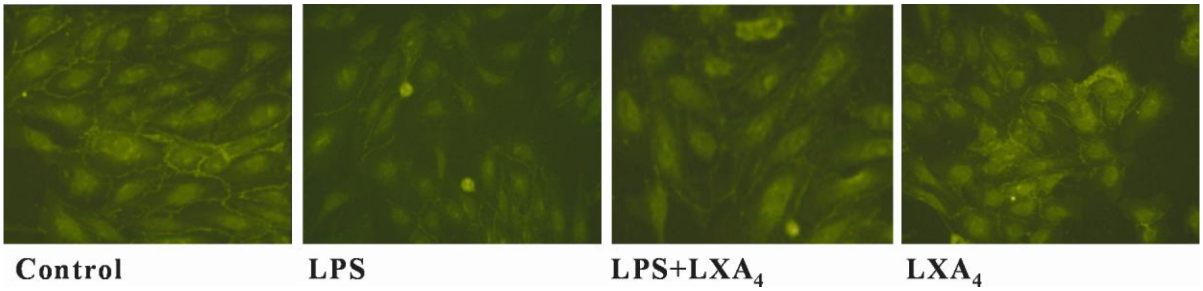

**FIGURE 4.** VE-cadherin protein expression detected by immunofluorescence. Representative images were from one of three separate experiments that yielded similar results.

### $LXA_4$ Inhibited LPS-Induced [Ca2+]i Elevation and TRPC1 Expression

Calcium is critical for the maintenance of cell-cell junctions[18]; thus, we next measured $[Ca^{2+}]$i in HUVECs to explore the possible mechanism under which $LXA_4$ could maintain VE-cadherin and β-catenin expression. As shown in Fig. 5A, treatment of HUVECs with LPS for 6 h induced the increase of $[Ca^{2+}]$i for more than 50% that of control cells ($p < 0.01$). However, it was statistically lower in cells cotreated with $LXA_4$ ($p < 0.01$).

Transient receptor potential protein family C 1 (TRPC1) acts as a store-operated calcium entry channel (SOC) in the cell membrane, which regulates not only cellular calcium concentration, but also vascular permeability[19]. As shown in Fig. 5B, its mRNA expression increased obviously in cells treated with LPS, while in cells treated simultaneously by LPS and $LXA_4$, this abnormal up-regulation was inhibited significantly ($p < 0.05$).

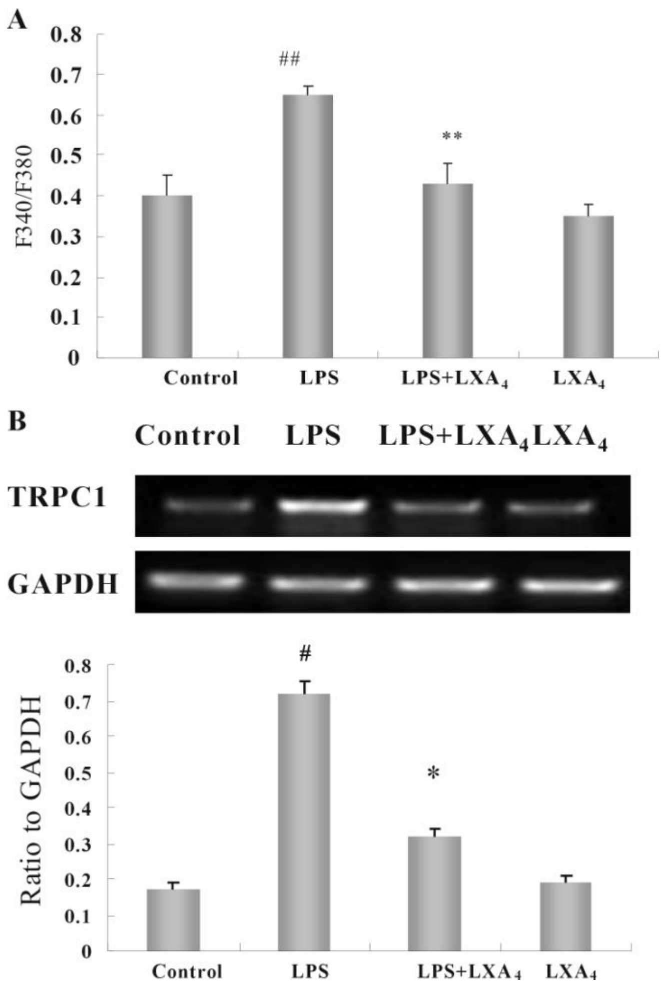

**FIGURE 5.** $[Ca^{2+}]i$ and TRPC1 expression in HUVECs under different conditions. (A) $[Ca^{2+}]i$ was measured with Fura-2 after cells were treated with LPS with or without $LXA_4$ for 6 h. Data are presented as ratio F340 to F380. All values are means ± SEM from at least three independent experiments. (B) Representative RT-PCR for TRPC1 is shown. Bar graphs indicate means ± SEM from at least three independent experiments; ##$p < 0.01$ compared to control cells; **$p < 0.01$ compared to cells treated by LPS alone; *$p < 0.05$ compared to cells treated by LPS alone.

### $LXA_4$ Blocked LPS-Induced ROS Generation in HUVECs

We further explored the effect of $LXA_4$ on cellular oxidative stress. Cells treated with LPS for 12 h showed ~1.5-fold increase of fluorescence intensity, indicating a significantly higher endogenous ROS formation (Fig. 6A). Cells cotreated with $LXA_4$ had significantly lower intensity compared with that in cells treated by LPS alone ($p < 0.05$).

The release of $H_2O_2$ from HUVECs is shown in Fig. 6B. At all three time points, $H_2O_2$ levels in the LPS group were much higher than those in the control group ($p < 0.05$) or the LPS + $LXA_4$ group ($p < 0.05$).

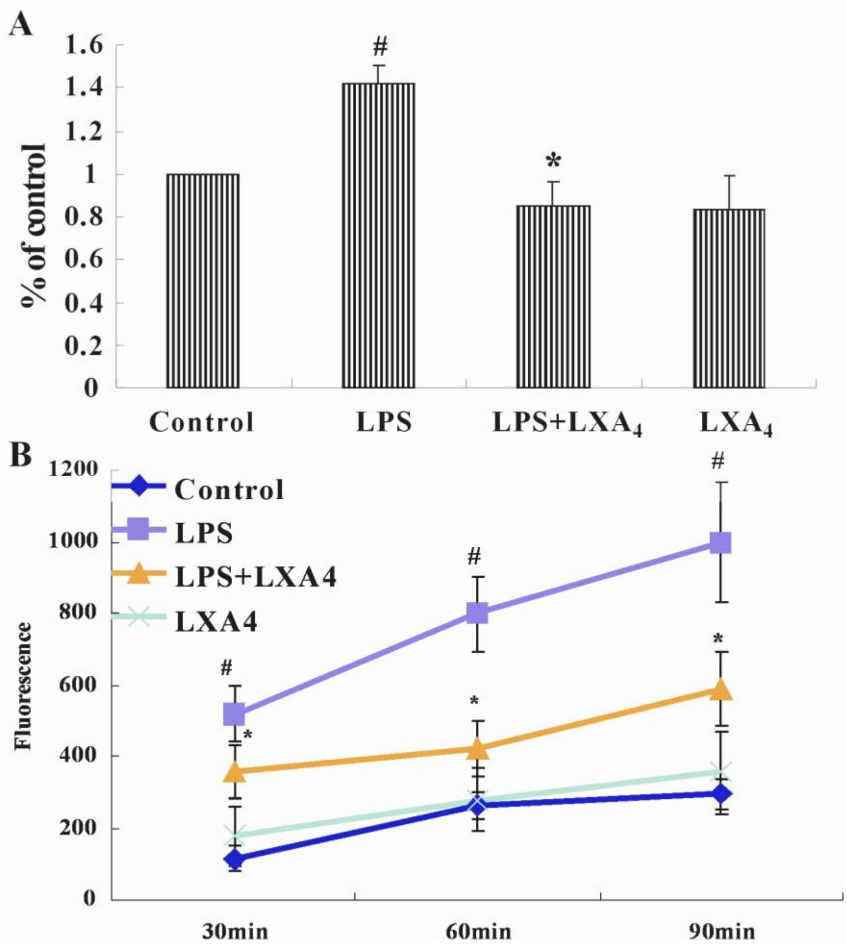

**FIGURE 6.** Effect of $LXA_4$ on cellular oxidative stress condition. (A) Intracellular ROS was evaluated with fluorometric assay employing DCFH-DA. (B) Extracellular $H_2O_2$ was measured with Amplex Red assay kit. #$p < 0.05$ compared to control cells. *$p < 0.05$ compared to cells treated by LPS alone.

### Effect of $LXA_4$ on the Antioxidant Response in HUVECs

In order to elucidate how $LXA_4$ inhibited ROS generation in LPS-stimulated HUVECs, Nrf2 protein level was measured. As shown in Fig. 7, it was down-regulated in LPS-treated cells, while up-regulated in cells cotreated with $LXA_4$ ($p < 0.05$).

### DISCUSSION

Ever since it was found that the disappearance of Evans blue dye from plasma was faster in patients with PE[20,21], increased capillary endothelial permeability was considered as a central event in the pathogenesis of PE[22]. Despite numerous studies pointing to the potent anti-inflammatory activities of LXs, there is a paucity of research examining their direct influence on the umbilical vessel. The purpose of this study was to determine the effect of $LXA_4$ on modulating permeability of HUVECs and the possible mechanism.

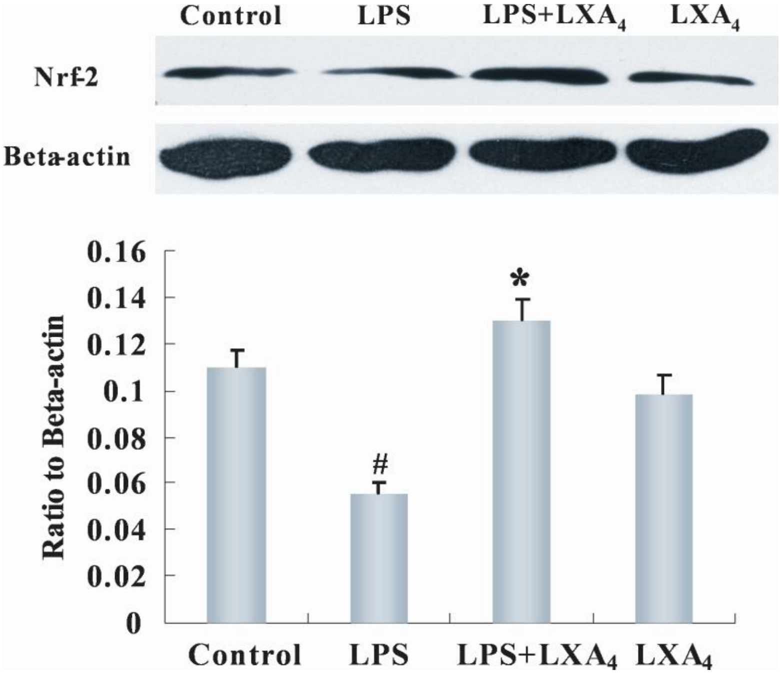

**FIGURE 7.** Effect of $LXA_4$ on Nrf-2 protein expression. Representative Western blot for Nrf2 is shown. Bar graphs indicate means ± SEM from at least three independent experiments; #$p < 0.05$ compared to control cells; *$p < 0.05$ compared to cells treated by LPS alone.

First, hyperpermeability of HUVECs was induced by administration of LPS, a commonly applied model in vascular leakage research[23]. Then it was found that $LXA_4$ strongly attenuated LPS-induced hyperpermeability, confirmed by both albumin permeability assay and morphological observation. ALXR is a well-known specific receptor of $LXA_4$ on a variety of cell types, including HUVECs[16]. In addition, Gronert et al. confirmed that LXs could bind with vascular endothelial-derived LTD4 receptor[24]. Furthermore, bioactions of LXs might also be mediated by some other distinct receptors[25]. In order to explore the signaling mechanisms responsible for the protective effect of $LXA_4$, the antagonist of ALXR was applied. Results indicated that $LXA_4$ inhibited LPS-induced hyperpermeability in HUVECs mainly through ALXR.

Cell permeability is regulated in part by the dynamic opening and closing of cell-cell AJs. In ECs, AJs are mainly composed of VE-cadherin, an endothelium-specific member of the cadherin family, which is linked through its cytoplasmic tail to the AJ proteins p120, β-catenin, and plakoglobin[17]. In the current study, $LXA_4$ obviously reversed down-regulation of both VE-cadherin and β-catenin caused by LPS. This might help to explain the prevention of $LXA_4$ on LPS-induced hyperpermeability. Although Serhan’s group previously found that LXs inhibited the neutrophil-mediated increase of vascular permeability in the mouse ear[26,27,28], and Ereso et al. recently also confirmed that $LXA_4$ attenuates microvascular fluid leakage during inflammation in rats[29], our study presented for the first time that $LXA_4$ had directly protective effect on AJs.

Regulation of endothelial permeability is a complex process that often depends on SOC[30]. Recent studies have identified TRPC1, a member of TRP family, as the essential component of the SOC in ECs[31]. The rise in $[Ca^{2+}]i$ through TRPC1 has been established as the initial pivotal signal that precedes EC cytoskeletal reorganization and the disassembly of VE-cadherin[18,31,32]. The fact that $LXA_4$ obviously attenuated the expression of TRPC1 in cultured HUVECs helped us to explain how it blocked the elevation of $[Ca^{2+}]i$ in LPS-stimulated cells. However, the details of the cellular signals on regulating

TRPC1-induced $Ca^{2+}$ influx and how these signals control endothelial permeability still need to be clarified. TRPC1 is also considered to be a link between cellular $Ca^{2+}$ overload and excessive production of ROS[33]. The latter could also affect EC permeability through the regulation of VE-cadherin and β-catenin[34].

In the endeavor to explore the mechanism involved in redox disturbance of PE women, the ischemic placenta is widely regarded as a key resource of ROS[1,2,3], but it should not be ignored that activated leukocytes, neutrophils in circulation, and ECs are also likely contributors to ROS accumulation[2,3,35,36,37]. These cells could be activated by factors derived from PE placentae, such as inflammatory and vasobioactive mediators, as well as ROS[38,39]. For example, when HUVECs were stimulated with plasma from women with PE, they has increased oxidative ability[40]. Our group previously presented the inhibitory effect of $LXA_4$ on ROS production in several disease models[5,6,7]. Nascimento-Silva et al. further reported that an aspirin-triggered $LXA_4$ analog suppressed ROS generation in ECs[41]. In the current study, $LXA_4$ strongly blocked LPS-triggered ROS production in HUVECs.

In addition to increased pro-oxidant activity, there is also evidence for a decreased antioxidant protective capacity in women with PE[42]. Nrf2 is a crucial transcription factor in regulating cellular antioxidant response. When cells are exposed to high levels of ROS, Nrf2 translocates into the nucleus and sequentially results in up-regulation of series downstream phase II enzymes, such as NAD(P)H:quinone oxidoreductase (NQO1) and heme oxygenase 1( HO-1), which have emerged as important mediators of antioxidant and cytoprotective action[43]. Combined with the data we recently published[44], we demonstrated that $LXA_4$ could trigger nuclear translocation of Nrf2 and promote the expression of Nrf2, NQO1, and HO-1. It has illuminated a possible mechanism of its antioxidant effect on HUVECs.

In summary, the present study indicates for the first time that $LXA_4$, at physiological concentrations, could prevent the LPS-induced EC hyperpermeability through maintaining the expression of VE-cadherin and β-catenin. It might involve modulation of $LXA_4$ on $Ca^{2+}$ and redox homeostasis. Although there are still deep gaps to fill, especially about the underlying mechanism, our data might also contribute to the potential therapeutic value of LXs for PE and other vascular endothelial hyperpermeability-associated diseases.

### ACKNOWLEDGMENTS

This work was supported by the National Natural Science Foundation of China (No. 81070510), the Natural Science Foundation of Wenzhou City, China (No. Y20090129), and the Doctoral Fund of the Ministry of Education of China (No. 200803430002).

## SUPPLEMENT

### Sequences of Primers and Reactions Conditions for RT-PCR

- VE-cadherin:
  5'- TGT TCA CGC ATC GGT TGT -3', 5'- GAA AGC GTC CTG GTA GTC G -3'
- Nrf2:
  5'- ATT GCC TGT AAG TCC TGG TCA -3', 5'- ACT GCT CTT TGG ACA TCA TTT CG -3'
- TRPC1:
  5'-AAT GTC GTG GTT GTG ATT GTG C -3', 5'-GTC TTT GGT GAG GGA ATG ATG T-3'
- GAPDH:
  5'-ACC AGC CCC AGC AAG AGC ACA AG-3', 5'-TTC AAG GGG TCT ACA TGG CAA CTG-3'

Amplification protocols included initial melt at 95°C for 5 min, melt at 94°C for 30 sec, anneal for 45 sec, and extend at 72°C for 30 sec, circle 35. Anneal temperature is 54°C for Nrf2 and TRPC1, while 57°C for VE-cadherin and GAPDH.

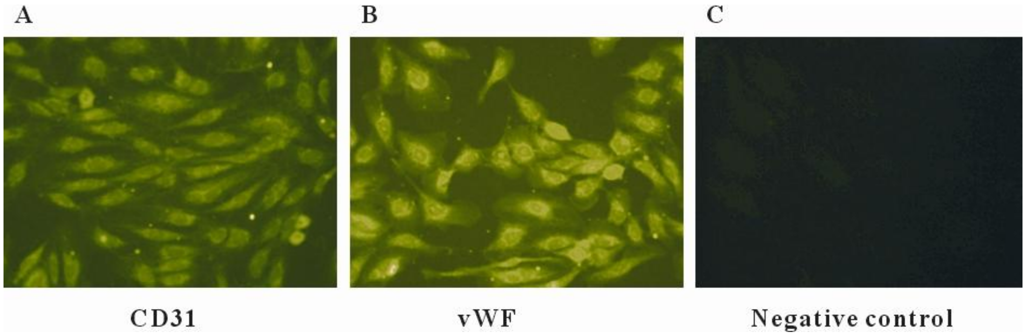

**SUPPLEMENTARY FIGURE 1.** Identification of primary HUVECs. Primary HUVECs were stained with antihuman CD31 (A) or von Willebrand factor (vWF) (B). Immunofluorescence assay showed that ≥95% cells isolated and cultured were positive. Rat lung fibroblasts stained with antirat vWF served as negative control (C).
